# Impact of Two Antibiotic Therapies on Clinical Outcome and Gut Microbiota Profile in Liver Transplant Paediatric Candidates Colonized by Carbapenem-Resistant *Klebsiella pneumoniae* CR-KP

**DOI:** 10.3389/fcimb.2021.730904

**Published:** 2021-12-14

**Authors:** Sabrina Cardile, Federica Del Chierico, Manila Candusso, Sofia Reddel, Paola Bernaschi, Andrea Pietrobattista, Marco Spada, Giuliano Torre, Lorenza Putignani

**Affiliations:** ^1^ Division of Gastroenterology, Hepatology and Nutrition, Bambino Gesù Children’s Hospital, IRCCS, Rome, Italy; ^2^ Multimodal Laboratory Medicine Research Area, Unit of Human Microbiome, Bambino Gesù Children’s Hospital, IRCCS, Rome, Italy; ^3^ Department of Diagnostic and Laboratory Medicine, Unit of Microbiology and Diagnostic Immunology, Unit of Microbiology, Bambino Gesù Children’s Hospital, IRCCS, Rome, Italy; ^4^ Division of Abdominal Transplantation and Hepatobiliopancreatic Surgery, Bambino Gesù Children’s Hospital, IRCCS, Rome, Italy; ^5^ Department of Diagnostic and Laboratory Medicine, Unit of Microbiology and Diagnostic Immunology, Unit of Microbiomics and Multimodal Laboratory Medicine Research Area, Unit of Human Microbiome, Bambino Gesù Children's Hospital, IRCCS, Rome, Italy

**Keywords:** liver transplantation, paediatric, microbiota, carbapenem-resistant *Klebsiella pneumoniae* (CR-KP), *Enterobacteriaceae*

## Abstract

Colonization by multidrug-resistant (MDR) organisms in liver transplant (LT) candidates significantly affects the LT outcome. To date, consensus about patient management is lacking, including microbiological screening indications. This pilot study aimed to evaluate the impact of carbapenem-resistant *Klebsiella pneumoniae* (CR-KP) colonization in LT paediatric candidates to enable optimal prevention and therapeutic strategies that exploit both clinical and microbiological approaches. Seven paediatric patients colonized by CR-KP were evaluated before and until one-year post LT. At the time of the transplant, patients were stratified based on antibiotic (ATB) prophylaxis into two groups: ‘standard ATB’ (standard ATB prophylaxis), and ‘targeted ATB’ (MDR antibiogram-based ATB prophylaxis). Twenty-eight faecal samples were collected during follow-up and used for MDR screening and gut microbiota 16S rRNA-based profiling. Post-transplant hospitalization duration was comparable for both groups. With the exception of one patient, no serious infections and/or complications, nor deaths were recorded. A progressive MDR decontamination was registered. In the ‘standard ATB’ group, overall bacterial richness increased. Moreover, 6 months after LT, *Lactobacillus* and *Bulleidia* were increased and *Enterobacteriaceae* and *Klebsiella* spp. were reduced. In the ‘targeted ATB’ group *Klebsiella* spp., *Ruminococcus gnavus*, Erysipelotrichaceae, and *Bifidobacterium* spp. were increased 12 months after LT. In conclusion, both antibiotics prophylaxis do not affect nor LT outcomes or the risk of intestinal bacterial translocation. However, in the ‘standard ATB’ group, gut microbiota richness after LT was increased, with an increase of beneficial lactic acid- and short-chain fatty acids (SCFA)-producing bacteria and the reduction of harmful *Enterobacteriaceae* and *Klebsiella* spp. It could therefore be appropriate to administer standard prophylaxis, reserving the use of ATB-based molecules only in case of complications.

## Introduction

The wide, often improper, use of antibiotics over the past few decades has led to the onset of bacterial resistance to antibiotics, which is becoming a global emergency (Tran et al., 2019). Infections caused by multidrug-resistant (MDR) bacteria have a relevant epidemiological value as both nosocomial and community-acquired infections, but also a clinical significance due to the morbidity and mortality that are highly associated with MDR (Colomb-Cotinat et al., 2016).

In solid organ transplantation (SOT), the incidence of bacteraemia due to Gram-negative bacteria, particularly enteric bacteria such as *Klebsiella pneumoniae* (KP), has significantly increased over time (Singh et al., 2004; Macesic et al., 2018). In endemic areas, the incidence of 1%-18% of carbapenem-resistant- (CR-) KP infections in SOT recipients is similar when comparing liver, kidney, lung, and heart transplants, with mortality reaching 60% (Pouch and Satlin, 2017). A multicentre study on transplanted adult patients who were carrying a CR-*Enterobacteriaceae* infection (CR-EI), showed a survival rate of 72%, with a median interval of 50 days since CREI to *exitus* one year after liver transplant (LT) (Huprikar et al., 2016). In comparison, the survival rate of the overall LT patients during the same time was higher than 88% (Adam et al., 2018; Kwong et al., 2020). The majority of these studies focused on adult LT recipients (Hand and Patel, 2016; Ferrarese et al., 2018) while in a paediatric population they are starting to appear (Del Chierico et al., 2018; Sun et al., 2021).

In paediatric age patients, biliary atresia (BA) represents the main indication for LT (Adam et al., 2018; Kwong et al., 2020). Most patients with BA undergo Kasai’s portoenterostomy after diagnosis, which improves survival, although it is burdened by complications such as ascending cholangitis (Cazares et al., 2017). Patients with BA are characterized by specific gut microbiota, likely due to an alteration of the bile flow with an inverse microbial richness proportional to the degree of cholestasis and a reduced abundance of *Enterobacter* (Tessier et al., 2020). Baek et al. showed that the most prevalent pathogens implicated in cholangitis-related infections are *Enterobacteriaceae* family members as *Escherichia coli*, *Enterobacter cloacae*, and KP (Baek et al., 2020). The gut microbiota represents a large reservoir of MDR bacteria (Gargiullo et al., 2019). In this environment, MDR bacteria can transfer their resistance genes to commensals increasing the pathogenic potential of the microbiota (Baron et al., 2018). This is particularly relevant for frail patients, especially when antibiotic therapies may select MDR bacteria already present in their gut microbiota (Rice, 2008; Biliński et al., 2016). Nevertheless, an eubiotic gut microbiota guarantees protection from gut colonization by MDR microbes through colonization resistance mechanisms (Ubeda et al., 2013), by competing bacteria for space and trophic resources, or by generation of bactericidal factors (Kim et al., 2017). MDR opportunistic pathogens may translocate across the intestinal barrier or contaminate skin and other body sites through catheter or intravenous lines (Lübbert et al., 2014), causing infections and even sepsis (van Schaik, 2015). Moreover, SOT recipients colonized by MDR bacteria may serve as important reservoirs of nosocomial transmission across hospital settings (Pereira and Uhlemann, 2016). Therefore, the monitoring of commensal microbiota alteration during antibiotic (ATB) treatment can assist in preventing and in treating adverse events occurring during antibiotic therapies, especially in SOT recipients.

In this context, this pilot study aimed to estimate the impact of MDR colonization on a small cohort of paediatric LT patients. Clinical evaluation, MDR bacteria monitoring, and gut microbiota profiling were evaluated to design optimal prevention and therapy strategies for these patients.

## Materials and Methods

### Study Population

We performed a longitudinal case study, enrolling paediatric patients affected by chronic liver disease or with other indications for LT with evidence of intestinal colonization by MDR bacteria.

From April 2015 to April 2018, the Paediatric Unit of Hepatology and Liver Transplantation Centre at Bambino Gesù Children’s Hospital (OPBG) of Rome, Italy, performed 92 LT procedures. As per hospital protocol, infectious diseases’ screening panels were performed before entering the transplant waiting list, including culture- and molecular-based microbiological assays. All patients were screened for KP by rectal swabs before LT. Seven out of 92 patients, regardless of being asymptomatic, resulted in being colonized by CR-KP. Since diagnosis, CR-KP intestinal colonization was regularly monitored, during a time-course up to 12 months since LT. Moreover, clinical course, including surgical and infectious complications, in the pre-, during and post-transplantation period was monitored. The clinical features of the colonized patients are reported in **Table 1**.

**Table 1:** Clinical features of the seven CR-KP colonized patients who underwent liver transplant (LT).

Feature	Values
Median age at admission (min and max values)	1.6 years (0.43-6.80 years)
Gender	1/7 male (14%); 6/7 female (86%)
Geographical origin	Russia (14%); Greece (42%); Romania (14%); Italy (29%)
Underlying liver disease	Biliary atresia (100%); Previous Kasai’s portoenterostomy procedure (86%)
Colonizing MDR microorganism	CR-KP *(bla*_KPC_) (100%)
Living donor graft	(71%)
Hospitalization days after LT, (average ± SD)	30.29 ± 10.33
Survival at 1 year after-LT	100%

Before the administration of perioperative ATB prophylaxis, the enrolled patients were stratified into two groups: ‘standard ATB group’, in which 3 patients received a standard ATB prophylactic therapy according to the OPBG protocol; and ‘targeted ATB’ group, in which 4 patients received an MDR antibiogram-based ATB prophylaxis.

Written informed consent was obtained from the patient’s parents. The study was conducted in accordance with the Declaration of Helsinki. The protocol was approved by Bambino Gesù Children’s Hospital Ethics Committee (Protocol Number 1538).

### Microbiological Assays

Rectal swabs were inoculated on MacConkey medium (Biomerieux, Marcy l’Etolie, France) and meropenem at 10 µg/ml was added. The identification of bacterial isolates was performed by MALDI-TOF MS (Seng et al., 2009). According to EUCAST guidelines (www.eucast.org), the isolates of Enterobacterales with meropenem MIC >0.5 µg/ml were submitted to a synergistic effect, modified Hodge tests (Centers for Disease Control and Prevention (CDC), 2009) and real-time PCR Xpert Carba-R (Cepheid, Sunnyvale, CA, USA). The *bla* gene encoding for *Klebsiella pneumoniae* carbapenemase (*bla*_KPC_), New Delhi Metallo-β-lactamase (*bla*_NDM_), Verona Integron-encoded Metallo-β-lactamase (*blaVIM*), oxacillinase (*bla*_OXA-48-48-181-232_) and imipenenase Metallo-β-lactamase (*bla*_IMP-1_) were tested.

### Gut Microbiota Profiling

Stool samples were collected from each patient before LT (pre), 1 month (1M), 6 (6M), and 12 months (12M) after LT. Each sample was immediately frozen at -80°C until treatment.

DNA was manually extracted from each sample using QIAmp Fast DNA stool mini kit (Qiagen, Germany) (Illumina, San Diego, CA) (Romani et al., 2020). The bacterial library was obtained by the amplification of 16S rRNA variable regions V3–V4 (~460 bp) (primer pair: 16S_F 5′-TCG TCG GCA GCG TCA GAT GTG TAT AAG AGA CAG CCT ACG GGN GGC WGC AG-3′ and 16S_R 5′-TC TCG TGG GCT CGG AGA TGT GTA TAA GAG ACA GGA CTA CHV GGG TAT CTA ATC C-3′) described in MiSeq rRNA Amplicon Sequencing protocol (Illumina, San Diego, CA). The PCR reactions were performed using 2x KAPA Hifi HotStart ready Mix (KAPA Biosystems Inc., Wilmington, MA, USA). Negative (absence of template) and positive (standard DNA) controls were introduced to monitor and exclude eventual external and internal contaminations. DNA amplicons were cleaned-up by AMPure XP beads (Beckman Coulter Inc., Beverly, MA, USA). The indexing PCR was performed using Illumina Nextera adaptor-primers (Illumina). Each library was cleaned-up by 50 ul of AMPure XP beads, quantified by Quant-iT™ PicoGreen^®^ dsDNA Assay Kit (Thermo Fisher Scientific, Waltham, MA), and diluted to the final concentration of 4 nM. Finally, all samples were pooled and sequenced on an Illumina MiSeqTM platform (Illumina). Raw sequences were analyzed by QIIME 1.9.1 software (Caporaso et al., 2010). Reads were clustered into Operational Taxonomic Units (OTUs) at 97% identity by UCLUST (Edgar, 2010) against the Greengenes 13.8 database (DeSantis et al., 2006). Analyses of alpha- and beta-diversity were performed by QIIME 1.9.1 software. Statistical tests (Mann Whitney, Wilcoxon, Benjamini-Hochberg tests) on OTUs relative abundances were computed by SPSS v. 20 software (IBM statistics).

Linear Discriminant Analysis (LDA) effect size (LEfSe) was used to identify OTUs with significantly different abundances between groups (Segata et al., 2011). The alpha value of 0.05 and an effect size threshold of two were used to identify the significant microbial taxa associated with each patient’s group.

All raw sequencing reads are available at the NCBI BioProject database (PRJNA741471) (https://www.ncbi.nlm.nih.gov/bioproject/).

## Results

### Patient Characteristics

The seven colonized patients (6/7 females, median age of 1.6 years), coming from different European countries (71.4% foreigner, 28.6% Italian), were admitted to the Paediatric Unit of Hepatology and Liver Transplantation Centre, already CR-KP positive. All patients were affected by BA, and six of them underwent Kasai’s portoenterostomy procedure before admission to our Centre (**Table 1**). After confirmation of CR-KP colonization, the use of antibiotics was avoided as for routine protocol, except for those patients with acute bacterial infections. All infection sites were monitored by culture-based procedures (*i.e*., blood, urine, etc.).

In only one patient, CR-KP-induced sepsis occurred before the LT, with simultaneous positivity of pharyngeal swab and urine samples. The infection was effectively treated with tigecycline and gentamicin, according to the antibiogram profile, resulting in persistent negative blood cultures regardless of positive stool cultures until LT. The LTs were performed from living donors in 5/7 cases. All patients underwent standard immunosuppressive regimen with basiliximab, and steroids’ *boli* plus oral tacrolimus continued as monotherapy from day 5 post-LT.

At the time of LT, patients were grouped based on antibiotic prophylaxis. Three patients, were treated by a usual antibiotic protocol based on the association of ampicillin and second-generation cephalosporin, were grouped in the ‘standard ATB’ group. Four patients, treated with antibiotic prophylaxis designed according to antibiotic sensitivity for CR-KP, were grouped in the ‘targeted ATB’ group (**Table 2**). As per our protocol, antibiotic prophylaxis is first administered in the surgical operating room and continued for 5 days if the patient was stable without any sign of sepsis. Systemic antifungals are used only in selected patients (*e.g.*, acute liver failure, patients with cystic fibrosis or on dialysis, re-transplant, etc.).

**Table 2:** Antibiotics used during liver transplantation in ‘Targeted ATB’ group.

Patient	Oral therapy	Intravenous therapy
1	Colistin	Colistin and Amikacin
2	Colistin and Gentamicin	Gentamicin and Colistin
3	–	Tigecycline and Gentamicin
4	Colistin and Gentamicin	Colistin and Amikacin

For both groups, the average post-transplant hospitalization period was comparable, 26.67 days for the standard group *vs* 33 days for the targeted ATB group (*p*>0.05). No deaths were recorded during the entire follow up. Except for *Pneumocystis jirovecii* pneumonia prophylaxis with trimethoprim/sulfamethoxazole for 6 months after transplantation, none received any further ATB treatment. The LT-related infectious, surgical complications, and rejection episodes were recorded immediately after LT (within the first month), and during long-term follow-up (from the first to the twelfth month) (**Table 3**).

**Table 3:** Observed post-transplant infections and clinical complications.

Patient	Infections (site, months post-LT)	Major surgical complications (months post-LT)	Acute rejection (months post-LT)	Hospital stay (days post-LT)	CR-KP colonization (timing of decontamination, months)
**‘Targeted ATB’ group**					
1	EF (peritoneal fluid, 1)	n.d.	yes (6)	41	4
2	CMV (blood, 1)	n.d	yes (1)	33	5
3	CMV (blood, 1)	Biliary stenosis (2)	n.d.	27	24
	SE (peritoneal fluid, 1)				
4	HHV 6 (blood, 1)	n.d.	yes (6)	31	8
	VRE (peritoneal fluid, 1)				
**‘Standard ATB’ group**					
1	CP and EF (peritoneal fluid, 1)	Biliary leakage with fistula (1)	n.d.	44	1
2	n.d.	Biliary stenosis (4)	n.d.	21	2
3	Adenovirus (blood, 1)	Gastrointestinal bleeding (6)	yes (1)	15	1
	EBV (blood, 6)		yes (6)		

CMV, Cytomegalovirus; HHV6, Human Herpes Virus 6; VRE, Vancomycin-Resistant Enterococci; LT, liver transplant; CR-KP,carbapenem-resistant Klebsiella pneumoniae; EBV, Epstein- Barr virus; SE, Staphylococcus epidermidis; EF, Enterococcus faecalis; CP, Candida parapsilosis; n.d., not detected.

For the ‘targeted ATB’ group, in the first month after LT, three systemic viral infections (cytomegalovirus [CMV], Human Herpes Virus 6 [HHV6]), and three peritoneal fluid bacterial infections (*Enterococcus*, *Staphylococcus*, Vancomycin-resistant Enterococci, [VRE]), were registered and successfully treated with specific therapy (**Table 3**). No surgical complications and only acute graft rejection were reported. In two of these four patients, CR-KP was isolated in the peritoneal fluid, urine, and pharynx, after LT. After six months, two episodes of acute graft rejection were registered, while in the following 11 months, one CR-KP infection triggered by dilatation of biliary stenosis was reported.

For the ‘standard ATB’ group one viremia episode (*i.e*., Adenovirus), two peritoneal fluid infections from *Enterococcus* and *Candida parapsilosis*, one biliary leakage with fistula formation, and one acute graft rejection occurred (**Table 3**). Moreover, one Epstein-Barr virus (EBV) infection was reported; one biliary stenosis and one episode of gastrointestinal bleeding were registered after 4 and 6 months, respectively. A further acute graft rejection occurred 6 months after LT (**Table 3**).

During the entire observational period, a progressive MDR faecal decontamination was detected in 6 out of 7 children. In particular, the patients who received ‘standard ATB’ incurred faecal decontamination 1.33 ± 0.58 months from the LT; while the patients who received the ‘targeted ATB’, obtained faecal decontamination in 10.25 ± 9.32 months. No statistical difference between faecal decontamination periods was observed.

In total, we registered a single serious infection, sepsis induced by CR-KP in one child of the ‘targeted ATB’ group 2 months post-LT. This patient suffered a similar septic episode before LT and, interestingly, after 2 years post-LT was still colonized by MDR bacteria, despite being asymptomatic.

### Gut Microbiota Ecology and Profiling in ‘Targeted’ and ‘Standard ATB’ Groups

**Figure 1:**
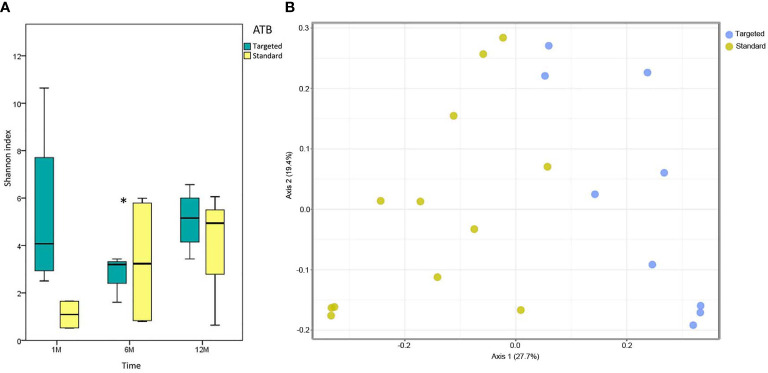
Microbiota ecology. **(A)** Whisker box plots of Shannon index of ‘targeted ATB’ and ‘standard ATB’ groups are reported for 1M, 6M and 12M points of the follow-up. The boxes display the minimum, first quartile, median, third quartile, and maximum of Shannon index for each group. Star indicates the statistically significant comparison at 6 months (M) (*p* value<0.05). **(B)** Principal Coordinates Analysis (PCoA) plot of ‘targeted ATB’ and ‘standard ATB’ groups for 1M, 6M and 12M points of the follow-up. The plot shows the first two principal axes for PCoA using unweighted UniFrac algorithm.

One month after LT, a reduction of microbiota richness was observed for the ‘standard ATB’ group. Six months after LT, a statistical increment of microbiota richness in the ‘standard ATB’ group was observed; while 12 months after LT, the microbiota richness resulted for both groups being comparable (**Figure 1**).

**Figure 2:**
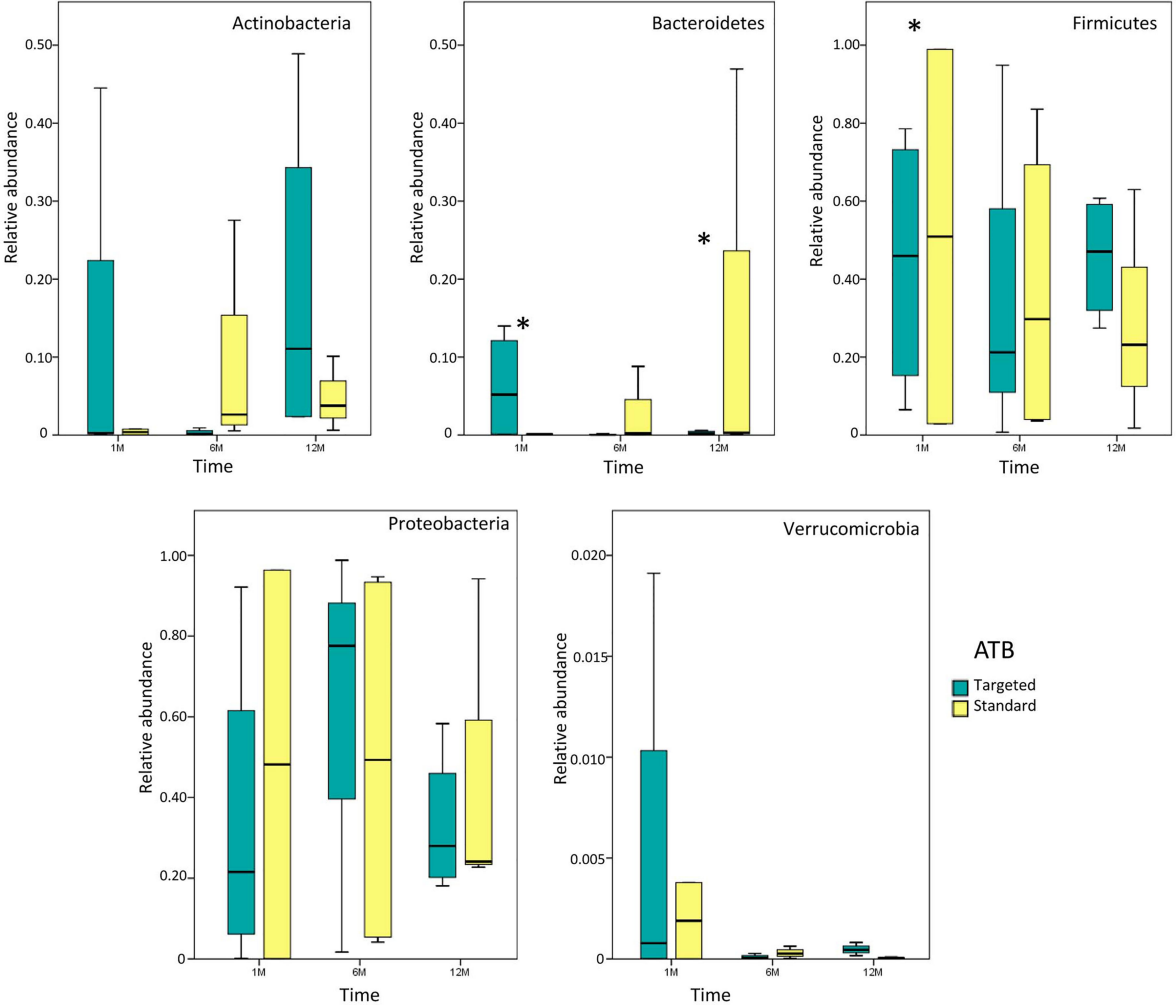
Whisker box plots of phylum distribution. Relative abundances of the main phyla for both ‘targeted ATB’ and ‘standard ATB’ groups are reported for 1M, 6M and 12M points of the follow-up. The boxes display the minimum, first quartile, median, third quartile, and maximum of phyla relative abundances for each subgroup. Stars indicate the statistically significant comparisons obtained by Mann Whitney test (*p* value<0.05).

Analyzing the gut microbiota composition at the phylum level, at 1 month after LT, Bacteroidetes results were higher in the ‘targeted ATB’ group compared to the ‘standard ATB’ group. At 12 months the pattern was inverted (*p*<0.05). Firmicutes were increased in the ‘standard ATB’ group 1 month after LT compared to the ‘targeted ATB’ group (*p*<0.05) (**Figure 2**).

**Figure 3:**
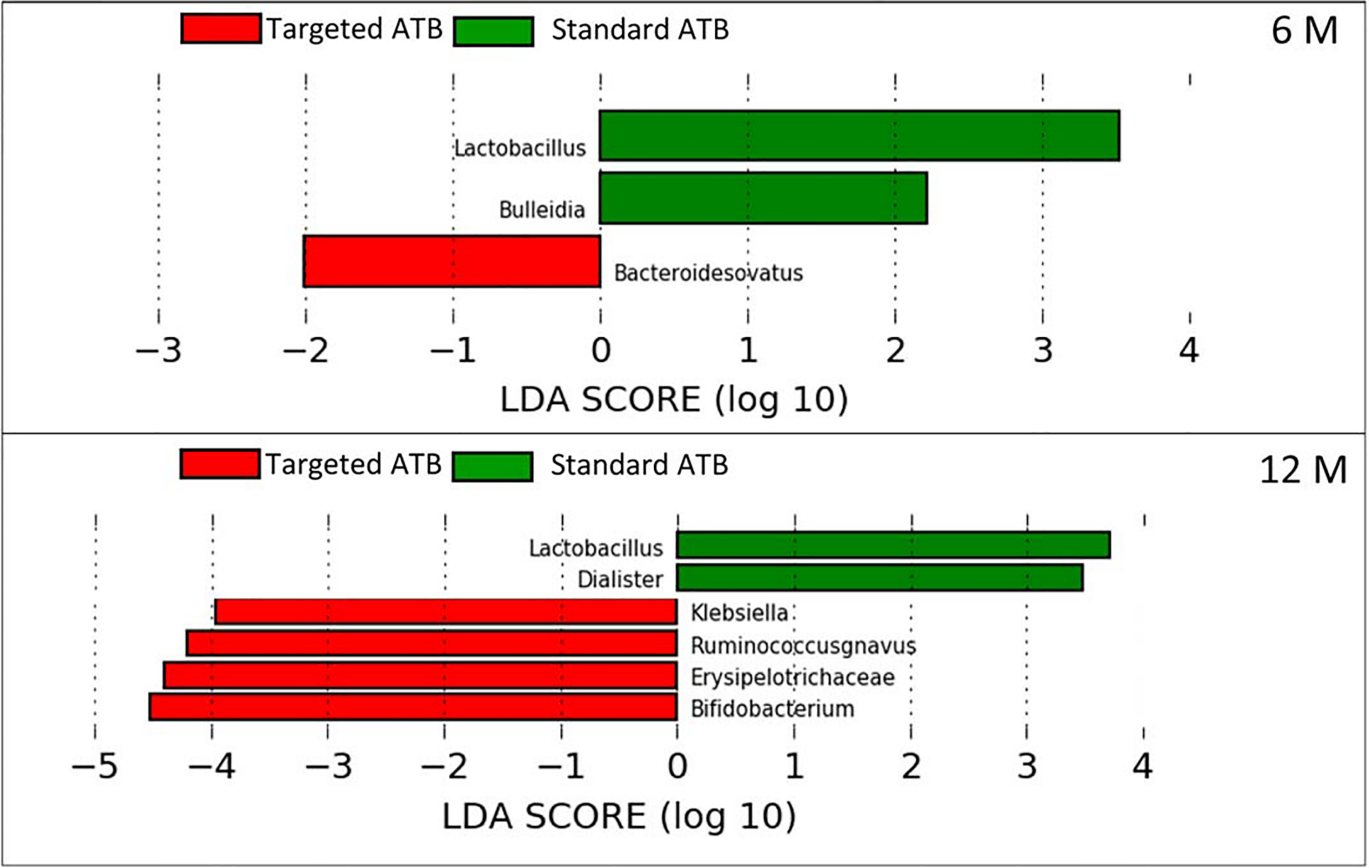
Linear Discriminant Analysis Effect Size (LEfSe) for the ‘targeted ATB’ and ‘standard ATB’ groups. The graphs display the differentially expressed OTUs in the ‘targeted ATB’ and ‘standard ATB’ groups, ranked by effect size performed at 6 and 12 months after LT.

Comparing the microbial content at the genus/species level, there were no statistical differences between the two groups one month after LT. After 6 months, *Bacteroides ovatus* distribution was increased in the ‘targeted ATB’ group, while *Lactobacillus* spp. and *Bulleidia* were increased in the ‘standard ATB’ group (**Figure 3**). At 12 months, *Lactobacillus* spp. and *Dialister* spp. Were increased in the ‘standard ATB’ group, while *Klebsiella* spp., *Ruminococcus gnavus*, *Erysipelotrichaceae*, and *Bifidobacterium* spp. increased in the ‘targeted ATB’ group (**Figure 3**).

### Evaluation of *Enterobacteriaceae* and *Klebsiella* spp. Persistence in the ‘Targeted’ and ‘Standard ATB’ Groups

**Figure 4:**
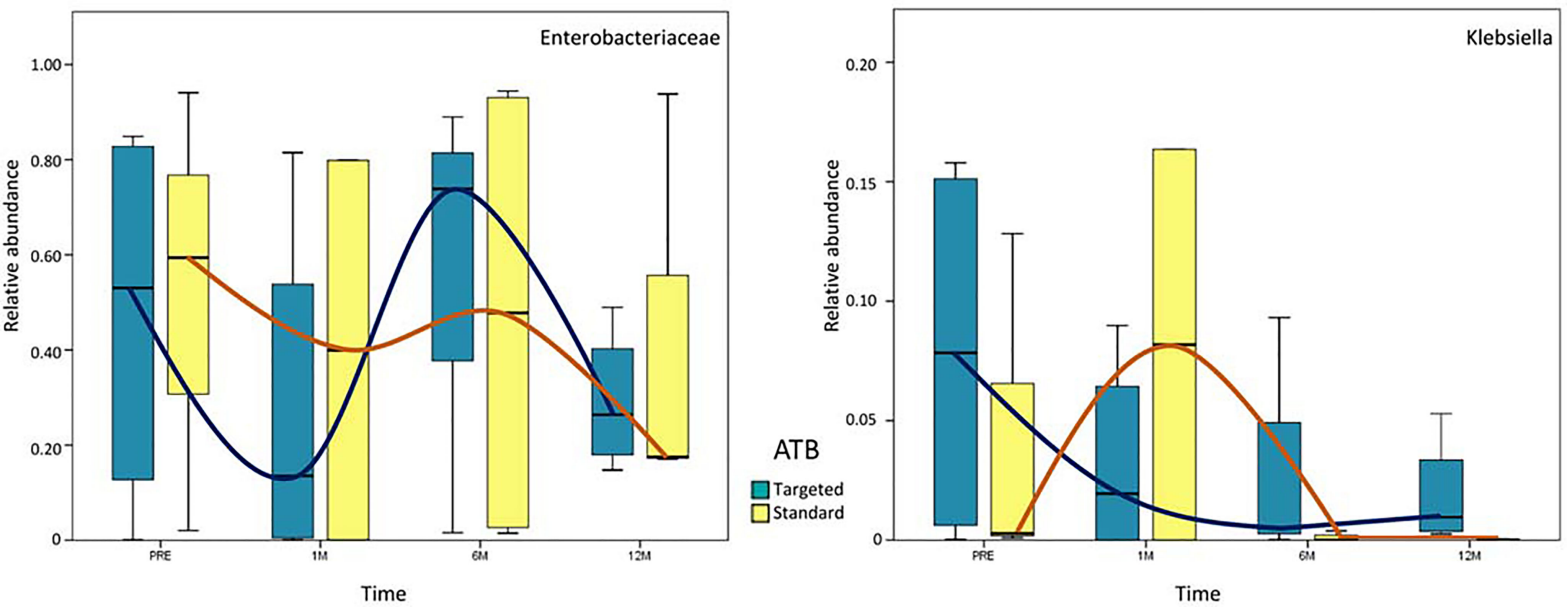
Whisker box plots of *Enterobacteriaceae* and *Klebsiella* spp. distribution. Relative abundances of *Enterobacteriaceae* and *Klebsiella* spp. are reported at each time-point for both ‘targeted ATB’ and ‘standard ATB’ groups. The boxes display the minimum, first quartile, median, third quartile, and maximum of *Enterobacteriaceae* and *Klebsiella* spp. relative abundances. The blue and orange lines evidence the trend of relative abundance for ‘targeted ATB’ and ‘standard ATB’ groups, respectively.

Analyzing the persistence of *Enterobacteriaceae* and *Klebsiella* spp., no statistically significant variation was observed. However, *Enterobacteriaceae* were very high (around 60% of the entire microbiota content) in both ATB groups in the pre-LT time-point. One month after LT, OTUs were more reduced in the ‘targeted ATB’ group compared to the ‘standard ATB’ group. After 6 months, *Enterobacteriaceae* increased prevalently in the ‘targeted ATB’ than in the ‘standard ATB’ group, until their reduction in both groups 12 months after LT (**Figure 4**). Regarding *Klebsiella* spp., in the ‘targeted ATB’ group, a constant progressive reduction of the relative abundance of this microorganism was observed along the follow-up time-course. In the ‘ATB standard’ group, an increment after 1 month and then the reduction towards minimal values from 6 months were observed (**Figure 4**).

## Discussion

The MDR screening program carried out at our Centre allowed us to perform early identification of MDR bacteria for either infected or colonized patients, thus avoiding the infection spreading. Moreover, this approach allowed for the implementation of “*ad hoc*” intervention strategies, especially for patients coming from European countries without any previous microbiological information, which represent most of the patients (71.4%). Furthermore, through this screening, we were able to distinguish MDR microbe “carrier” patients before transplant from those who contracted the infection from the graft and/or in the period following the transplant. This information is useful especially in split liver and multi-organ transplants from a single donor for which the early intercommunication amongst recipient centers about the possible status of the organ’s carrier may be helpful for patient management following transplant (Mularoni et al., 2015; Errico et al., 2019). Awareness of the high vulnerability of these patients, especially in the early stages, leads to the wide use of antibiotics, but in MDR-colonized patients, it is not yet well established whether it is necessary to empirically resort to targeted antibiotics or use standard prophylactic therapy.

In 2018, the group for the ‘Study of Infection in Transplantation of the Spanish Society of Infectious Diseases and Clinical Microbiology and Spanish Network for Research in Infectious Diseases’ did not recommend a surgical prophylaxis regimen different from the local standard for adult recipients colonized with CP-*Enterobacteriaceae* before LT (Aguado et al., 2018). Moreover, previous studies have been conducted only for MDR infection in LT during outbreaks, over limited time periods, or with a retrospective modality (Birgand et al., 2013; Feldman et al., 2013; Lübbert et al., 2014; Sun et al., 2021).

The prophylaxis of paediatric patients affected by MDR bacteria with targeted antibiotics still represents an open avenue and it is important to increase consciousness of the risks of over-treatment. For this reason, different ATB prophylactic regimens in homogeneous groups of MDR bacteria affected paediatric patients need to be assessed. Our multi-disciplinary approach allowed us to evaluate the reduction of antibiotic mismanagement during the transplant waiting list. Moreover, we also evaluated the impact of post-operative complications without affecting the rates of morbidity and mortality (up to 12 months) of our patients’ compared to non-MDR patients, especially in the first phases after transplant, where intense immunosuppression is required. Moreover, in our cohort, post-transplant complications including reoperation or surgery complications, rejection incidence, and infections, were similar in both groups, both in the immediate post-transplant and in the medium and long follow-up period. This evidence suggests that MDR microbe ‘targeted ATB’ prophylaxis associated with LT prevents neither the onset of complications related to the transplantation itself, nor the risk of bacterial translocation from the gastrointestinal tract, nor the post-LT hospitalization length. In the case of sepsis, we used a combination of antibiotics rather than a monotherapy with a good outcome, as recommended by scientific literature (Bassetti et al., 2018), suggesting that targeted-ATB therapy represents a valid choice in the case of effective treatment and not for prophylaxis. Of course, the use of other therapies (antibiotic, antiviral, and antifungal) in the post-transplant period may potentially affect the outcome but, since the complications are similar in the two groups, we consider additional therapies as part of the normal management of transplant patients.

In our previous study, performed in a single case study, we proposed microbiota profiling in the clinical management of these transplant patients. Our evidence opened a new point of view that may guide clinicians on the choice of the appropriate antibiotic strategies and monitor the MDR colonization and microbiota eubiotic reversion after MDR colonization and ATB treatment insults (Del Chierico et al., 2018). The identification of high-risk patients by microbial signatures may allow for tailored interventions and an improvement in LT outcomes (Annavajhala et al., 2019).

Our results demonstrate a faster increase in microbiota richness in the medium term after standard ATB therapy, suggesting a more rapid restoration of gut microbiota eubiosis compared to the ATB targeted therapy. Moreover, the standard treatment produced a positive effect in the increment of the beneficial Actinobacteria and Bacteroidetes and the reduction of potentially harmful Proteobacteria compared to the ATB targeted treatment, even in the medium- and long-term. In particular, the microbiota of patients treated by standard ATB therapy was enriched in beneficial microorganisms like *Lactobacillus* spp., *Bulleidia*, and *Dialister*, which are natural producers of antimicrobials and short chain fatty acids (SCFAs) (Umu et al., 2017). On the contrary, the microbiota of the patients treated by targeted therapy was enriched in the highly immunogenic *Erysipelotrichaceae* family (Kaakoush, 2015), in the inflammatory polysaccharide producer, such as *Ruminococcus gnavus*, and in the opportunistic pathogen as *Klebsiella* spp. (Martin and Bachman, 2018). Furthermore, both treatments reduced the abundance of *Enterobacteriaceae* at the end of the follow up, but the standard therapy seemed to anticipate this effect at 6 months, while the targeted one was affected only at 12 months. In addition, in standard ATB prophylaxis *Klebsiella* spp. was completely eradicated, while in targeted ATB prophylaxis a slight and progressive reduction of it was observed, even maintaining positivity until 12 months.

## Conclusion

Despite the small number of patients – due to the fact that hepatic transplantation in paediatric age patients is less common than in adults and the presence of MDR bacteria in these patients, which, fortunately, does not cover a wide range of cases – this study offers an innovative discussion of the management of paediatric MDR patient candidates to LT, by addressing multiple aspects of this problem. To the best of our knowledge and despite the limitations outlined below, this is the first study analyzing MDR bacteria colonization and microbiota profiling in a paediatric population undergoing LT, comparing two antibiotic prophylactic regimens (standard vs targeted) in the management of these patients. In our case series, colonization with MDR bacteria did not represent a contraindication for LT; the presence of MDR bacteria at the time of transplant did not compromise the prognosis and almost all patients had progressive decontamination, mainly after being discharged. Early MDR microbe identification and active surveillance may allow clinicians to establish a specific strategy of prevention and targeted treatment in case of complications, avoiding broad-spectrum antibiotic overuse. The routine use of a ‘targeted ATB’ therapy during transplantation does not seem to bring benefits either from a clinical or microbiological point of view.

These gut microbiota profiling results are noteworthy in the description of the impact of each therapy on this complex ecosystem, especially in the presence of CR-KP colonization. In particular, standard therapy was effective in early decontamination of CR-KP, restoring gut microbiota eubiosis, beneficial bacteria growth, and the spontaneous reduction of *Klebsiella* spp.

Based upon the results of this study, we suggest that for MDR microbe colonized patients it would be appropriate to use a standard protocol of prophylaxis for LT management, reserving the use of targeted antibiotics only in case of serious complications.

## Data Availability Statement

The dataset presented in this study can be found online: https://www.ncbi.nlm.nih.gov/PRJNA741471.

## Ethics Statement

The protocol was approved by the Bambino Gesù Children’s Hospital Ethics Committee (Protocol Number 1538). Written informed consent to participate in this study was provided by the participants’ legal guardian/next of kin.

## Author Contributions

Conceptualization, GT. and LP. Methodology, SR and FC. Formal analysis, FC. Investigation, SC, FC, SR, MC, PB, AP, and MS. Resources, LP. Data curation, SC and FC. Writing-original draft preparation, SC and FC. Writing—review and editing, GT, LP, MS, SC, and FC. All authors contributed to the article and approved the submitted version.

## Funding

This work was supported by the Ministry of Health (Ricerca Corrente 201802G004314 assigned to LP).

## Conflict of Interest

The authors declare that the research was conducted in the absence of any commercial or financial relationships that could be construed as a potential conflict of interest.

## Publisher’s Note

All claims expressed in this article are solely those of the authors and do not necessarily represent those of their affiliated organizations, or those of the publisher, the editors and the reviewers. Any product that may be evaluated in this article, or claim that may be made by its manufacturer, is not guaranteed or endorsed by the publisher.
